# Voltage-Disturbance Identification and Fast Transfer Control of an IGCT-Based Hybrid Solid-State Transfer Switch

**DOI:** 10.3390/s26216862

**Published:** 2026-10-30

**Authors:** Bin Zhao, Ruoxi Liu, Hanqi Dai, Zexi Chen, Dawei Wang, Pu Wang

**Affiliations:** 1 Beijing Key Laboratory of Power Supply Assurance Technology and Application for Resilient Power Grids in Megacity, Electric Power Research Institute, State Grid Beijing Electric Power Company, Beijing 100070, China; 120232101121@ncepu.edu.cn (B.Z.); liuruoxi@sgcc.com.cn (R.L.); daihanqi@sgcc.com.cn (H.D.); wangdawei@sgcc.com.cn (D.W.); wangpu@sgcc.com.cn (P.W.); 2 School of Electrical and Electronic Engineering, North China Electric Power University, Beijing 102206, China

**Keywords:** Hybrid Solid-State Transfer Switch, integrated gate-commutated thyristor, fast transfer control, deep neural network, fault diagnosis

## Abstract

Voltage disturbances in power distribution networks can interrupt power supply to sensitive loads, thus requiring fast and reliable power switching. For low-voltage distribution networks, this study proposes a hybrid solid-state transfer switch based on IGCT, which integrates a magnetically controlled switch and combines voltage disturbance identification and fast switching control methods. The proposed method processes the acquired three-phase voltage waveforms through a hierarchical control architecture. A transient triggering strategy based on dq transform is used to detect abnormal voltage conditions and initiate the switching process, while a deep neural network model with multi-scale feature fusion identifies disturbances from transient voltage features. The identification method was evaluated under simulated noise conditions, and the switching performance was verified on a physical prototype platform. At a signal-to-noise ratio of 20 dB, the proposed model achieved a fault identification accuracy of over 95% under the tested operating conditions. Under single-phase full voltage interruption conditions, the switching time of the prototype was 4.91 ms, approximately 62.2% shorter than the 12.99 ms measured for the tested commercial thyristor-based solid-state transfer switch (Socomec). Under three-phase full voltage interruption conditions, the switching time of the prototype was 6.80 ms. The results show that the proposed hybrid solid-state transfer switch integrates voltage disturbance identification and fast switching control functions, providing a feasible solution for improving the power supply continuity of sensitive loads.

## 1. Introduction

With the continuous deepening of the construction of new power systems, the proportion of new energy connected to the power generation end is increasing, and the number of nonlinear and impulsive loads on the user side is increasing. The frequency and complexity of power quality disturbances (PQDs) are becoming increasingly higher. Taking the Beijing distribution network as an example, the number of voltage sag events recorded during the “14th Five-Year Plan” period alone reached more than 80,000. Voltage sags, voltage interruptions and harmonics are very likely to cause equipment malfunctions or shutdowns, resulting in huge economic losses [1,2]. According to the ITIC (CBEMA) curve released by the Information Technology Industry Council (ITI), the tolerance limit of sensitive loads such as solid-state electronic devices to power interruptions is usually in the millisecond range, requiring power supply switching to be completed within a very short time window, which puts forward more stringent requirements on power supply protection devices [3]. Against this background, the development of fast switching devices that can meet the power supply continuity of highly sensitive loads in modern industry has become one of the important research topics in the development of important power distribution protection equipment.

At present, the industry mainly uses automatic transfer switches (ATS), pure solid-state transfer switches (SSTS) and hybrid solid-state transfer switches (H-SSTS) to achieve dual power supply redundancy. Although ATS has the advantage of extremely low steady-state loss, it is constrained by mechanical inertia, and its action time is usually more than 50 ms. Moreover, it is prone to arcing when cutting off large currents. Further reductions in ATS transfer time require consideration of mechanical actuation, contact bounce, reliable source isolation, and load-dependent electrical transients. For motor loads, residual magnetic flux and continued rotor motion can sustain a residual voltage at the load terminals after the original supply is disconnected. Reconnection to the backup source with a substantial phase-angle or voltage-magnitude mismatch can produce high transient currents and electromagnetic torque stresses. Although a shorter interruption can limit motor deceleration and phase drift, it does not inherently eliminate the risk of out-of-phase reconnection. In particular, break-before-make interlocking prevents simultaneous connection of the two sources but does not ensure phase compatibility between the backup source and the residual load-side voltage. Consequently, the benefits of fast transfer should be evaluated together with the resulting electrical and mechanical stresses on the connected loads. The reported transfer times characterize the prototype under the tested conditions. Further validation is required to assess motor reconnection transients over a wider range of residual-voltage magnitudes, phase-angle differences, and loading conditions. Although pure SSTS offers an extremely fast response, its continuous conduction losses and thermal-management requirements can significantly compromise system reliability and economic viability in high-current distribution applications [4]. H-SSTS, which combines the advantages of both, has become the mainstream evolution direction for high-capacity fast switching. Comprehensive reviews have summarized the configurations, control strategies, transfer mechanisms, and application constraints of solid-state transfer switches [5,6]. Large-capacity prototypes have further demonstrated the feasibility of hybrid transfer architectures that combine low-loss mechanical current paths with fast semiconductor-assisted commutation [7]. However, most traditional H-SSTS are based on ordinary thyristors (SCRs), which require waiting for the current to cross zero and turn off, resulting in slow switching speed. Impulse-commutated SSTS schemes can actively shorten the turn-off process of conventional thyristors; however, they require an additional resonant commutation branch and coordinated triggering logic [8]. In 2023 Okilly et al. [9] developed a Triac-based static transfer switch using online voltage sensing and zero-crossing detection, and demonstrated a transfer time within one cycle in a 220 V, 60 Hz experimental system. In 2026, S.Y. Zhao et al. [10] proposed a hybrid resonant turn-off circuit for data center needs. It aims to drive the LC resonance through an auxiliary switch to force the SCR to turn off, achieving millisecond-level commutation and optimizing the volume of the auxiliary branch. However, it still requires complex commutation auxiliary logic in essence, and faces the inherent risks of a dramatic increase in system volume and control failure in medium- and high-voltage high-capacity applications. Because semi-controlled devices have always had limitations such as large commutation circuit size, complex logic, and applicability only to AC, researchers have been exploring the application of fully controlled power devices with self-turn-off capability in solid-state switches. In 2006, Zheng Jianyong’s team at Southeast University [11] verified the working characteristics and fast switching performance of a hybrid automatic transfer switch based on insulated-gate bipolar transistors (IGBTs) through experiments. In 2014, Lu Sheng zhi et al. [12] further developed a fast dual-power transfer switch based on IGBT, verifying that its power supply gap was only 1/4 cycle. In 2025, G.Y. Zhou et al. [13] pointed out through comparative analysis that using IGBT to replace thyristors can shorten the longest switching time from 11 ms to 4.2 ms. Although the IGBT-based scheme has active turn-off capability and flexible control, there are still obvious physical bottlenecks in terms of withstanding large current surges and high voltage stress. Against this backdrop, the integrated gate commutated thyristor (IGCT), which combines the high current density of thyristors with the hard turn-off capability of fully controlled devices, has shown unique advantages in the field of high-power power electronics.

The power interruption time of the load under solid-state switching is the most critical indicator reflecting the switching performance, which is determined by the total switching time. The total switching time depends not only on the physical speed of the high-power switching devices and mechanical mechanisms, but also on whether the detection algorithm can sensitively and accurately identify faults in complex environments [14]. At the sensing level, the detection algorithm is the “nerve center” for H-SSTS to execute switching decisions. Power quality detection methods include various signal processing methods such as short-time Fourier transform, wavelet transform, S-transform, instantaneous dq transform, and Hilbert–Huang transform. Among them, the instantaneous dq transform is widely used because it can obtain power quality characteristics simply and quickly, and many researchers have made improvements based on it [15]. In 2018, Li Xinian et al. [16] regarded single-phase voltage as three-phase unbalanced voltage, used the instantaneous symmetrical component method to extract the positive sequence component, and finally realized single-phase voltage fault detection through dq transformation. This method simplifies the calculation process and requires less computing power. In 2020, Zhou Jun et al. [17] proposed the derivative-based αβ-dq transformation based on the derivative method to realize the rapid detection of single-phase voltage sag, and combined it with morphological filtering to achieve efficient noise reduction. In addition to dq-transformation-based approaches, selective harmonic extraction has been investigated for rapid voltage-sag detection under nonideal grid conditions [18]. However, from the perspective of engineering practice, the single threshold trigger logic is very easy to cause device malfunction due to insufficient feature extraction when facing non-stationary disturbances, transient harmonics and strong background noise. Especially under high current conditions, the non-synchronous loop closing caused by logic misjudgment will have disastrous consequences. Recent studies have increasingly combined time-frequency analysis, signal decomposition, and pattern-recognition techniques for PQD diagnosis [19]. In particular, EMD-derived intrinsic mode functions can be represented by statistical features for subsequent disturbance classification [20], while multi-domain feature fusion and deep neural models provide an effective means of identifying single and combined disturbances under different noise levels [21]. It can be seen that building a set of intelligent detection system with high recognition and strong robustness can most effectively improve the comprehensive performance of intelligent H-SSTS.

In summary, although the existing research has optimized the device topology and detection algorithm to a certain extent, few studies have explored the organic integration of the physical characteristics of power electronic devices and deep learning algorithms to form hardware and software co-optimization. Based on this, this paper develops a novel intelligent H-SSTS based on IGCT and fast magnetically controlled switch. First, by analyzing the arc voltage characteristics during contact opening, a topology of parallel connection of IGCT and magnetically controlled circuit breaker is proposed. This natural commutation method, which eliminates the need for auxiliary commutation circuits, enables arc-minimized rapid disconnection under high current. Second, a hierarchical control architecture consisting of dq transformation instantaneous triggering and intelligent diagnostic algorithms is constructed, enabling rapid detection of power faults and accurate classification of fault types. Finally, an H-SSTS prototype integrating the algorithm and topology is developed, and experiments verify the significant advantages of the proposed scheme in ultra-fast switching and intelligent decision-making.

## 2. Electrical Topology and Commutation Mechanism

### 2.1. Topology of the H-SSTS

**Figure 1:**
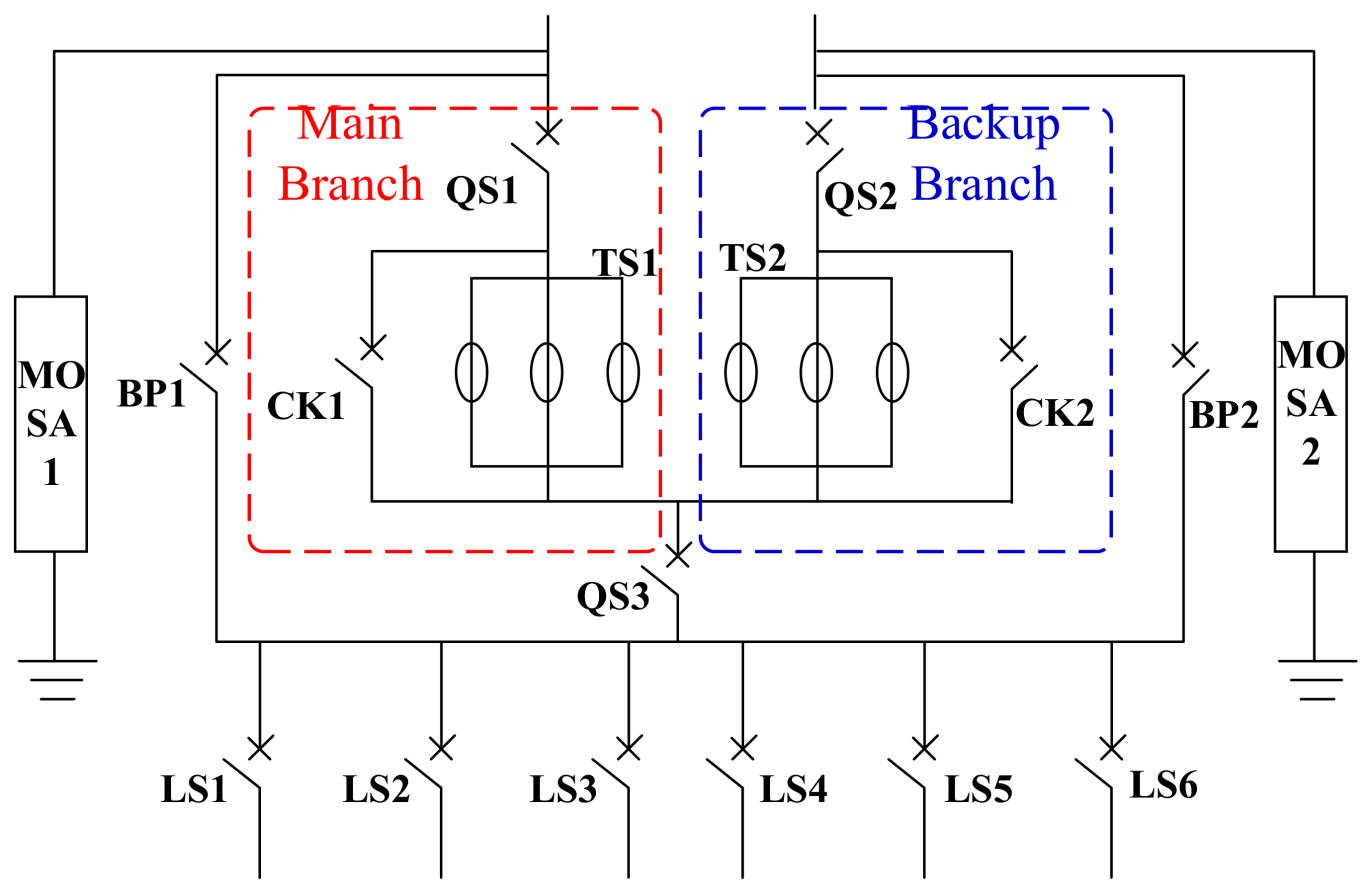
Electrical topology of the proposed dual-source H-SSTS.

The overall electrical topology of the intelligent hybrid solid-state transfer switch (H-SSTS) designed in this paper is shown in Figure 1. The system adopts a dual power supply configuration with main and backup power inputs, supplying power to the load side through six outgoing switches (LS1~LS6). The core switching unit of each power supply branch consists of a mechanical branch and a solid-state branch connected in parallel. Among them, the mechanical branch use fast magnetically controlled switches (CK1, CK2) driven by permanent magnet mechanisms (PMA). Compared with switches driven by traditional spring mechanisms, these magnetically controlled switches simplify the mechanical linkage and use electromagnetic force to drive the contacts directly, thereby shortening the operating time and improving coordination among the three phases. The solid-state branch (TS1, TS2) consists of multiple anti-parallel IGCT arrays. In the design, multiple IGCTs are connected in parallel to distribute the current stress under high current system and utilize their excellent di/dt tolerance to ensure the safety of devices during high current commutation.

The integrated incoming disconnect switches (QS1, QS2), outgoing disconnect switches (QS3), and manual maintenance bypass switches (BP1, BP2) in the topology meet on-site maintenance needs. When maintenance is required on the core IGCT module or magnetically controlled switch, the maintenance bypass is closed first to ensure continuous power supply to the load, and then the disconnect switches on the incoming and outgoing sides are opened. This physically isolates the core components from the energized circuit, ensuring the safety of the maintenance process. In addition, a metal oxide surge arrester (MOSA) is configured on the incoming side of the device to suppress grid surges and transient over-voltages caused by switching operations.

### 2.2. Analysis of the Natural Commutation Mechanism Driven by Arc Voltage

This subsection analyzes the current commutation process during the controlled disconnection of the main-source branch, specifically when a nonzero branch current remains following a transfer request triggered by a detected voltage disturbance. Before the transfer request, the mechanical switch CK1 is closed and carries the load current, while the parallel IGCT branch TS1 is blocked. The controller first applies a turn-on command to TS1 and then issues an opening command to CK1. Enabling TS1 prepares the commutation path; it does not imply that the load current immediately transfers away from the closed mechanical contacts.

As the contacts of CK1 separate under nonzero current, a short-duration vacuum arc develops. The resulting arc voltage drives the current from the mechanical branch into the enabled IGCT branch. This subsection focuses on that current-transfer interval, before the subsequent turn-off of TS1. Previous studies have shown that the vacuum-arc voltage and commutation-loop inductance significantly influence this process [22]. In the simplified equivalent circuit, the difference between the arc voltage and the IGCT forward voltage drop drives the increase in the IGCT-branch current. If the resistive voltage drop in the commutation loop is neglected, the current transfer rate can be expressed as:

(1) $$\frac{di_{\mathrm{TS}}}{\mathrm{dt}}=\frac{U_{\mathrm{arc}}(t)-V_{\mathrm{IGCT}}}{L_{\sigma }},$$

where *V_IGCT_* is the forward conduction voltage drop of the IGCT array, *U_arc_* is the dynamic arc voltage and *L_σ_* is the stray inductance of the commutation circuit. The prototype employs low-inductance multilayer busbar technology to optimize the physical structure, which reduces the stray inductance *L_σ_* and thus improves the current transfer rate. In a 380 V system, the vacuum arc voltage is typically stable between 20 V and 50 V, significantly higher than the approximately 2–5 V forward conduction voltage drop of the IGCT. This voltage difference ensures that the load current can be quickly and smoothly transferred from the mechanical contacts to the solid-state branch, and the mechanical contacts can also complete physical separation under low arc energy conditions. This not only avoids severe contact erosion but also lays the physical foundation for the prototype to achieve rapid half-cycle disconnection. After the current has transferred from CK1 to TS1, the control sequence proceeds to TS1 turn-off, verification of main-source disconnection, and connection to the available backup source through TS2. These subsequent stages belong to the overall transfer sequence described in Section 2.3 and are not represented by the commutation-rate model in Equation (1).

### 2.3. Switching Control Logic Flow

**Figure 2:**
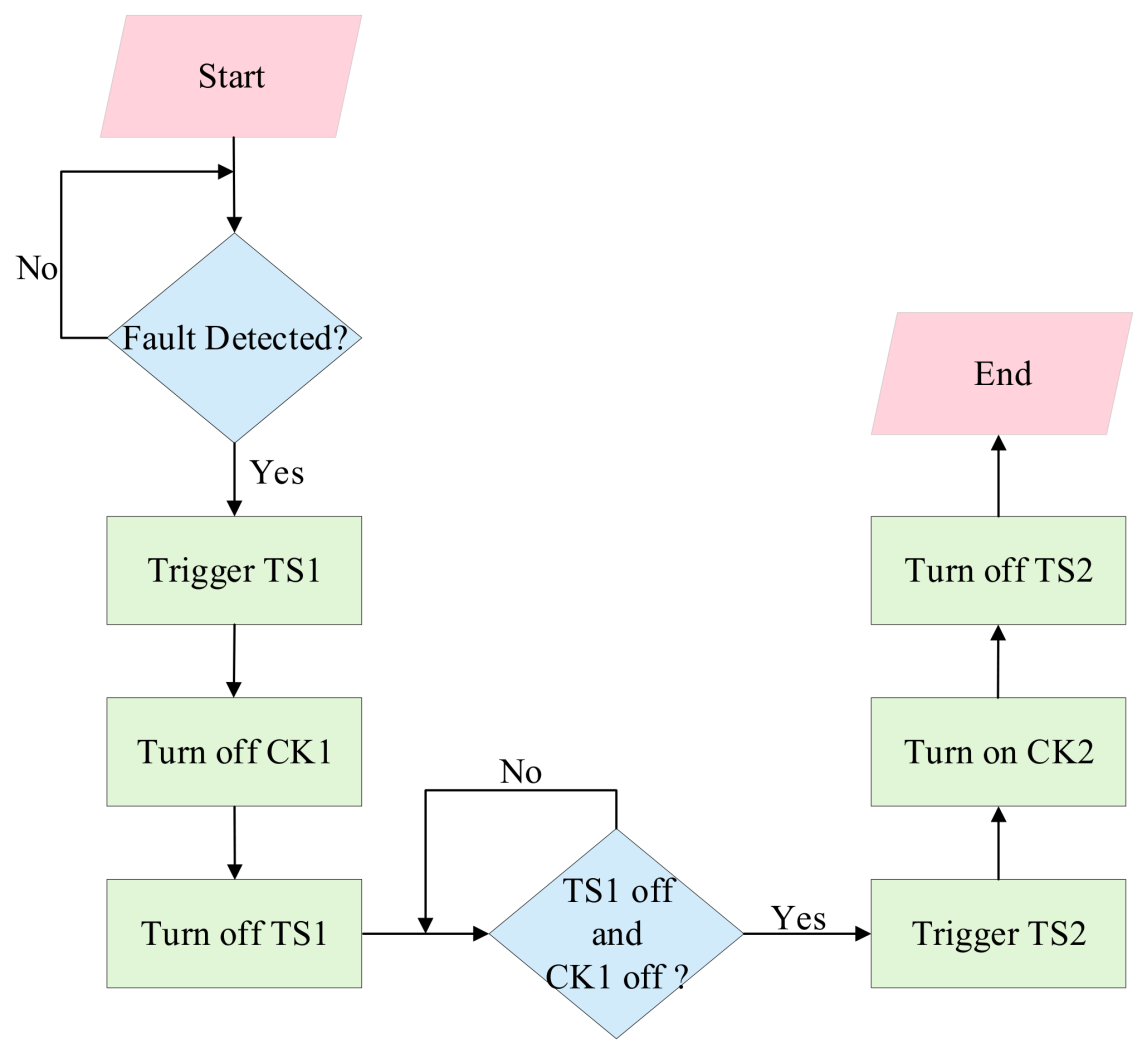
Flowchart of the switching control logic.

The switching control logic flow of the device is shown in Figure 2. Its core design principle is to ensure power supply continuity while maintaining absolute physical isolation between the main and backup power supplies. During steady-state operation, both the incoming disconnect switch and the main magnetic switch are closed. The load is powered by the main power supply via the magnetic switch, and the solid-state branches are in a blocked state to avoid losses.

The parallel IGCT branch TS1 assists current commutation and controlled interruption during the opening of CK1. Enabling TS1 before CK1 opens prepares an alternative current path, while the load current initially remains predominantly in the closed mechanical branch. As the CK1 contacts separate under nonzero current, the resulting arc voltage drives current commutation into TS1, reducing the current carried by the separating contacts. This arrangement is intended to limit arcing energy and contact erosion. TS1 is subsequently turned off to interrupt the main-source branch current. Its temporary turn-on therefore enables current takeover, whereas its subsequent turn-off completes electronic interruption. When the controller detects a switching command, the system immediately triggers the main solid-state switch TS1 to turn on, followed by the opening command of the magnetic switch CK1. This timing design aims to utilize the arc voltage generated when the contacts separate to quickly transfer the main circuit current to the solid-state branches. After confirming that TS1 has completed current takeover, the system immediately sends a shutdown signal to TS1, causing the main circuit to enter the disconnection determination stage.

After TS1 receives the shutdown command, the controller monitors and collects the instantaneous values of the three-phase current of the main branch in real time through high-frequency sampling. Considering sensor noise and calculation errors, the system only considers the main power supply physically disconnected after all three-phase currents have dropped below the preset zero current threshold, indicating that the arc has been completely extinguished and the solid-state devices have been blocked. This logical decision is crucial to the entire control sequence, fundamentally mitigating the risk of loop closure caused by asynchronous parallel connection of the main and backup power supplies.

A voltage sag on the main grid incomer does not imply that this incomer has been electrically disconnected. Enabling TS2 while CK1 or TS1 still provides a conducting path could unintentionally interconnect the two grid incomers, potentially causing circulating currents. Therefore, backup-source connection must follow verification of main-source disconnection and confirmation that the backup source satisfies the transfer conditions. Upon confirming the safe disconnection of the main circuit and that the backup circuit voltage meets requirements, the system immediately triggers the backup circuit solid-state switch TS2. This allows the load voltage to recover rapidly; at this stage, the backup source supplies the load through TS2, restoring the load voltage. To return to low-loss operation, the system then issues a closing command to the backup magnetic switch CK2. Considering the physical bounce of the mechanical contacts during closing, the system sets a fixed delay of 30 ms. After the contact bounce disappears and a stable mechanical current-carrying state is reached, the solid-state branch TS2 is finally deactivated, thus completing the full closed-loop switchover from fault response to steady-state power supply.

## 3. Intelligent Fault Diagnosis Algorithm and Simulation Analysis

### 3.1. Layered Perception and Collaborative Diagnostic Architecture

**Figure 3:**
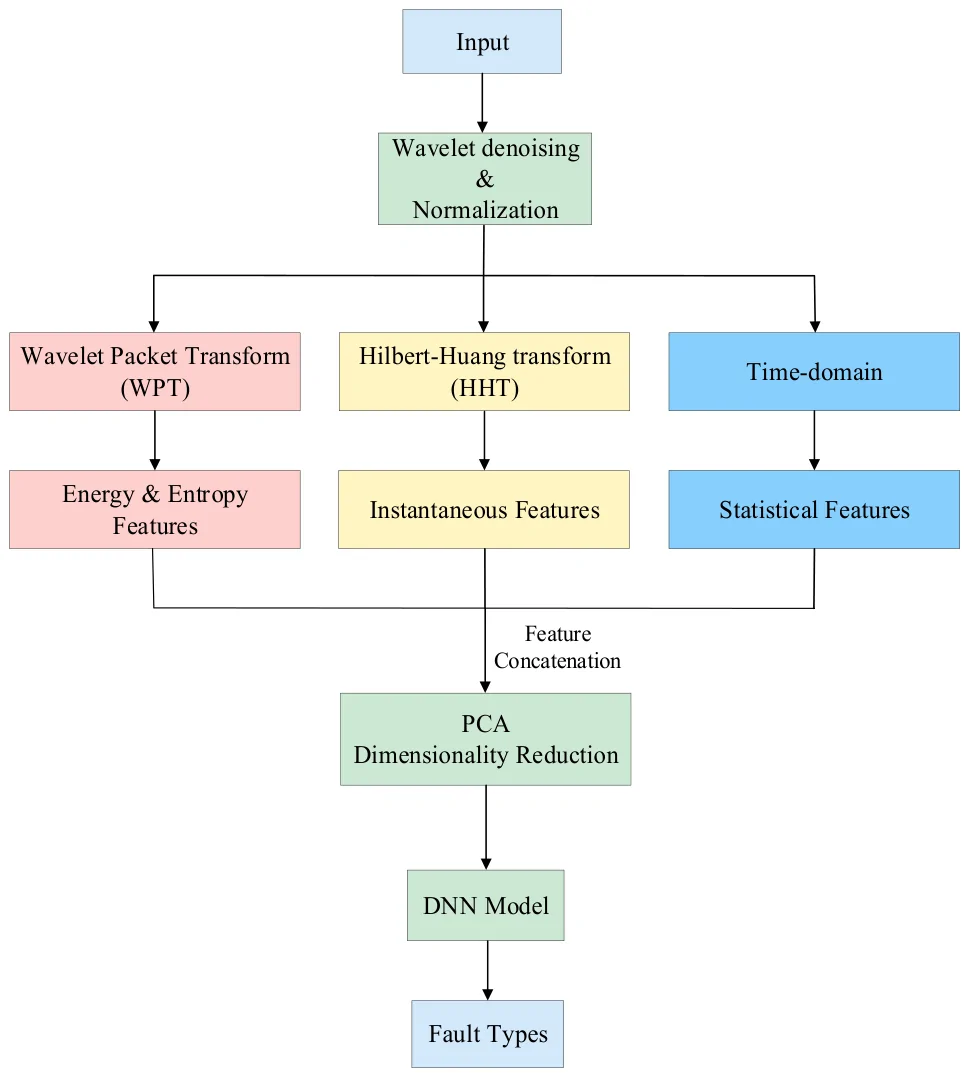
Flowchart of the intelligent identification scheme.

To enable SSTS to achieve both rapid switching and intelligent fault classification, this paper designs a multi-algorithm collaborative hierarchical sensing framework. This architecture splits the detection task into two paths: instantaneous triggering and deep diagnosis. The instantaneous triggering layer includes the Park algorithm based on dq transform and Fast Fourier Transform (FFT). On the one hand, the dq transform monitors the amplitude and phase of the three-phase voltage in real time, aiming to identify severe voltage sags, voltage swells, or short-circuit anomalies within an extremely short time of 3 ms. On the other hand, the FFT algorithm calculates the total harmonic distortion (THD) in the power grid in real time, and immediately issues a trigger command once the harmonic content exceeds the load tolerance threshold. The dq-based detection path and the DNN-based diagnostic path operate in parallel. The DNN runs on the CPU of a PC, with an inference time of approximately 4 ms on the tested platform. When the dq-based trigger criterion is satisfied, the controller initiates the transfer sequence without waiting for the DNN output. If the dq-based path does not generate a transfer request, the DNN classification result is evaluated against the corresponding transfer criteria to determine whether a transfer is required. In the voltage-sag and interruption experiments reported in this study, the dq-based path reliably detected the disturbances and triggered the transfer. Consequently, these transfers did not require waiting for the DNN result. For transfers that depend on DNN classification, however, the availability of that result affects the transfer-initiation time. Figure 3 shows the complete flowchart of the intelligent diagnostic algorithm from signal preprocessing and feature mining to model classification.

### 3.2. Multi-Scale Feature Extraction and Mathematical Derivation

For each terminal node at decomposition level (n), the node energy *E*_*j*,*n*_ is calculated using Equation (2a). It is then normalized by the total energy of all terminal nodes at the same decomposition level within the same analysis window, as expressed in Equation (2b). The Shannon entropy *S*_*j*,*n*_ is calculated using Equation (2c).

(2a) $$E_{j,n}=\sum_{k}{\left|d_{j,n,k}\right|}^{2},$$

Here, *n* denotes the decomposition level, *j* = 0, 1, …, 2*^n^* − 1 indexes the terminal nodes, and *k* indexes the wavelet packet coefficients within each node. The coefficient *d*_*j*,*n*,*k*_ is the *k*th wavelet packet coefficient of node *j* at level *n*. The quantities *E*_*j*,*n*_ and ${\tilde{E}}_{j,n}$ denote the unnormalized and normalized node energies, respectively. For a nonzero total energy, the normalized node energies lie between 0 and 1 and sum to unity. The normalized values ${\tilde{E}}_{j,n}$ are used as the wavelet packet energy features. In Equation (2c), *p_i_* denotes the *i*-th probability in the energy-based probability distribution used to calculate the Shannon entropy.

(2b) $${\tilde{E}}_{j,n}=\frac{E_{j,n}}{\sum_{m=0}^{2^{n}-1}E_{m,n}}$$

(2c) $$S_{j,n}=-\sum_{i}p_{i}\ln(p_{i}),$$

where *d*_*j*,*n*,*k*_ are the wavelet coefficients and *p_i_* is the energy probability distribution. In the formula, *d*_*j*,*n*,*k*_ are wavelet packet coefficients, and *p_i_* is the energy probability distribution. Here, the entropy feature is particularly sensitive to waveform disturbances caused by harmonic distortion or high-frequency flicker. The second approach uses the Hilbert–Huang transform (HHT). First, empirical mode decomposition (EMD) decomposes the non-stationary signal *x*(*t*) into several intrinsic mode functions (IMFs) and a residual component *r_n_*(*t*):

(3) $$x\left(t\right)=\sum_{i=1}^{n}c_{i}\left(t\right)+r_{n}\left(t\right),$$

where *c_i_*(*t*) represents the i-th IMF component obtained from the empirical mode decomposition. Then, the Hilbert transform of each IMF component is calculated:

(4) $${\hat{c}}_{i}(t)=\frac{1}{\pi }P.V.\int_{-\infty }^{+\infty }\frac{c_{i}(\tau )}{t-\tau }d\tau ,$$

where ${\hat{c}}_{i}\left(t\right)$ represents the Hilbert transform of *c_i_*(*t*), and P.V. represents the Cauchy principal value, used to handle the singularity of the integral at *τ* = t. Then, the analytic signal $z_{i}\left(t\right)=c_{i}\left(t\right)+j{\hat{c}}_{i}\left(t\right)$ is constructed, and the instantaneous amplitude of the *i*-th IMF component is calculated:

(5) $$a_{i}(t)=\sqrt{{c_{i}}^{2}(t)+{{\hat{c}}_{i}}^{2}(t)}.$$

This paper uses the mean and standard deviation of the first three IMF component amplitudes as feature values to effectively characterize the nonlinear features in electromagnetic transients. Finally, the standard deviation, peak value, and other indicators of the original data and the difference sequence are statistically analyzed, and these time-domain parameters are concatenated with the aforementioned features to construct a comprehensive fault identification feature vector.

### 3.3. Feature Dimensionality Reduction and Classification Network Design

To address the computational burden caused by feature redundancy in multi-scale feature extraction, Principal Component Analysis (PCA) is introduced for feature dimensionality reduction. First, a feature covariance matrix is constructed, and then eigenvalue decomposition is used to project the complex high-dimensional space onto mutually perpendicular principal component spaces. Multiple simulation experiments show that, while retaining 95% of the cumulative contribution rate, the feature dimension is adaptively compressed from 99 to 32 dimensions. This indicates that the selected features at each scale contain rich fault feature information, and the features are highly independent.

The classifier is a fully connected deep neural network (DNN) that takes the 32-dimensional WPT–HHT fused feature vector obtained after standardization and PCA as its input. It comprises three fully connected hidden layers containing 128, 64, and 32 neurons, respectively, followed by a seven-neuron output layer. All hidden layers use rectified linear unit (ReLU) activation functions. Dropout layers with a rate of 0.2 are placed after the first and second hidden layers. The output layer uses Softmax activation to produce class probabilities for seven operating states: normal operation, single-phase-to-ground fault (AG), phase-to-phase fault (AB), two-phase-to-ground fault (ABG), three-phase fault (ABC), open-circuit fault, and voltage sag/swell.

The network is trained using the Adam optimizer with an initial learning rate of 0.001, a batch size of 32, and a maximum of 100 epochs. Sparse categorical cross-entropy is used as the loss function for the integer-encoded class labels. Early stopping monitors the validation loss with a patience of 15 epochs and restores the weights corresponding to the lowest validation loss. A model-checkpoint callback also saves the model with the highest validation accuracy.

### 3.4. Simulation Result Verification and Performance Evaluation

To evaluate the classification performance of the proposed model, simulated voltage-disturbance signals were generated in Python 3.13 for seven operating states: normal operation, AG fault, AB fault, ABG fault, ABC fault, open-circuit fault, and voltage sag/swell. Two test datasets were constructed using the signal-generation procedure: a source test dataset and a generalization test dataset.

**Figure 4:**
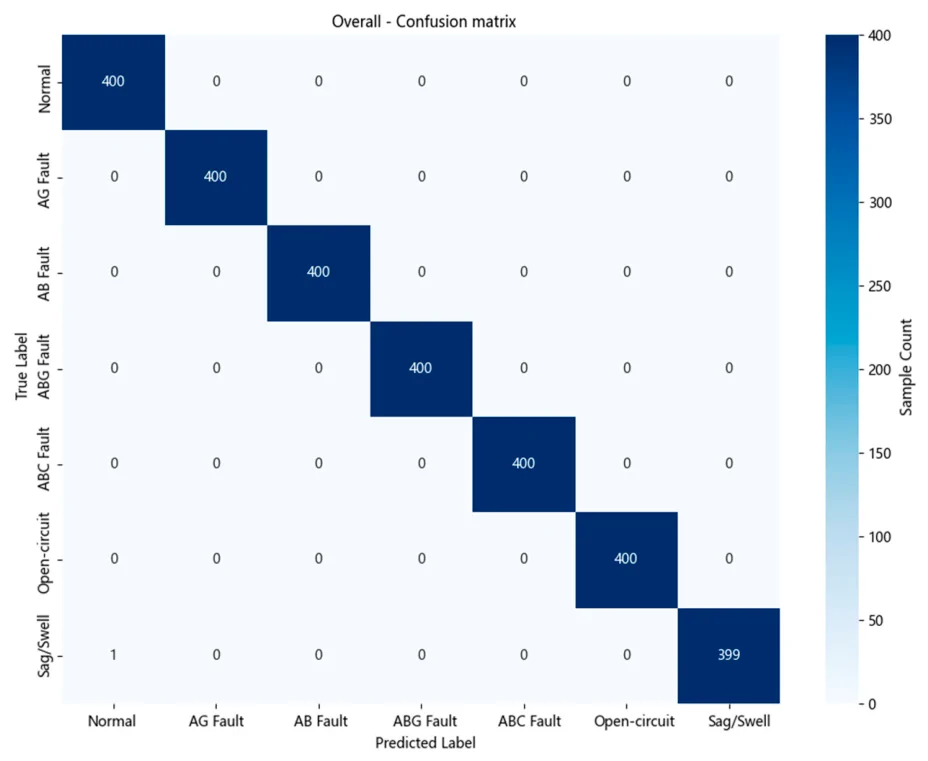
Overall confusion matrix for fault identification on the source dataset.

**Figure 5:**
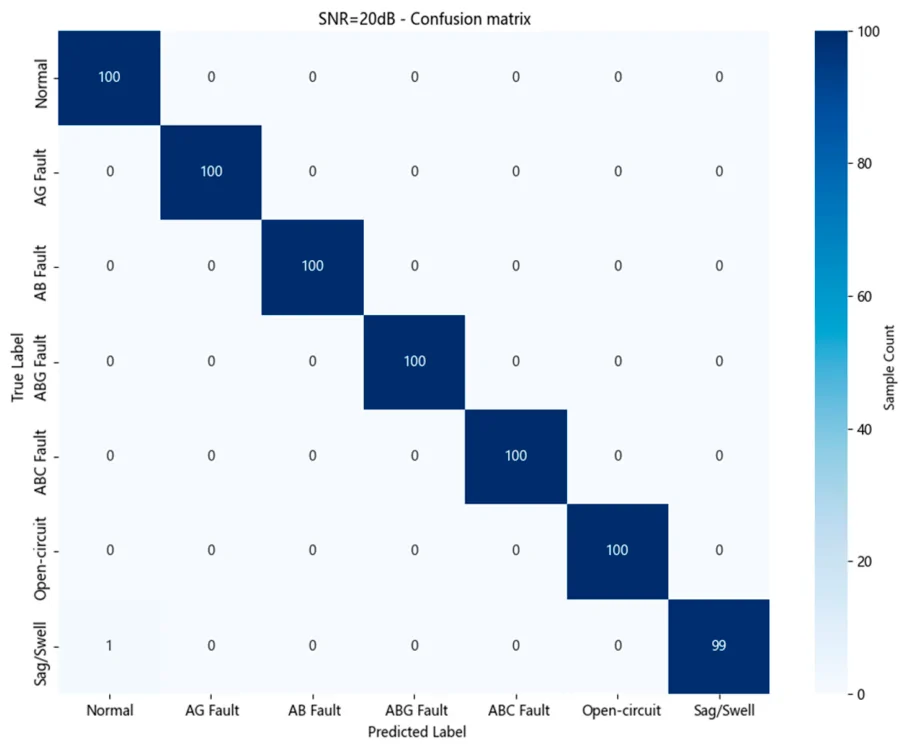
Confusion matrix for fault identification on the source dataset at SNR = 20 dB.

For the source test dataset, the disturbance-generation parameters were drawn from the same ranges as those used to generate the training dataset. For the generalization test dataset, selected parameter ranges were expanded beyond those used for training, while the class definitions remained unchanged. Thus, the source test dataset evaluates classification performance within the training parameter ranges, whereas the generalization test dataset evaluates performance over a broader operating range. Noise robustness was evaluated by adding Gaussian white noise at signal-to-noise ratios of 20, 30, and 40 dB. For each noise condition, both the source and generalization test datasets contain 100 samples per class, giving 700 samples per dataset per condition. The classification performance of the proposed model on the source dataset is shown in Figure 4 and Figure 5. The model achieved an overall accuracy of 100% under the noise-free, 40 dB, and 30 dB conditions. Even when the signal-to-noise ratio was reduced to 20 dB, the overall accuracy remained at 99.86%, indicating that the proposed model maintains high classification accuracy and strong robustness to noise.

**Figure 6:**
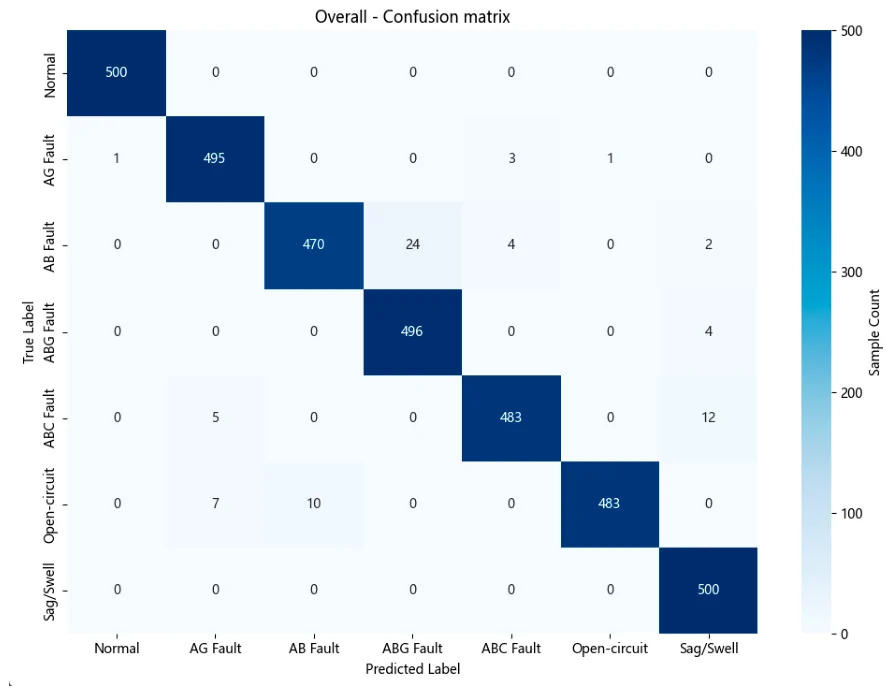
Overall confusion matrix for fault recognition on the generalization dataset.

**Figure 7:**
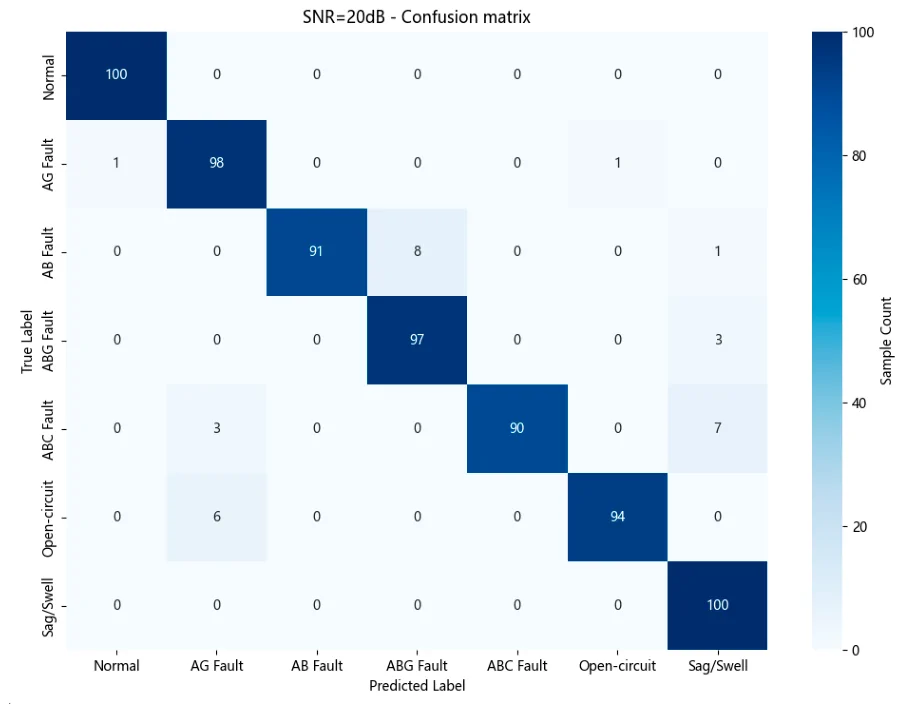
Confusion matrix at SNR = 20 dB for fault recognition on the generalization dataset.

The performance of the model on the generalization dataset is presented in Figure 6 and Figure 7 and summarized in Table 1. Compared with the source dataset, the fault signals in the generalization dataset exhibit a wider range of feature parameters, providing a more stringent evaluation of the model’s ability to identify previously unseen operating conditions. On the overall generalization dataset, the model achieved an overall accuracy of 97.91%, a macro recall of 97.91%, a macro F1-score of 97.91%, and a normal-class precision of 99.80%. At a signal-to-noise ratio of 20 dB, the overall accuracy and macro recall decreased to 95.71%, while the macro F1-score and normal-class precision were 95.72% and 99.01%, respectively. The confusion matrix indicates that most misclassifications occurred between phase-to-phase short-circuit (AB) and two-phase-to-ground short-circuit (ABG) faults. This may be attributed to their similar physical characteristics and the partial masking of zero-sequence information by high noise levels. Overall, the results demonstrate that the model maintains satisfactory classification performance and robustness under complex and noisy operating conditions.

The different classification indices for the two test sets under overall and 20 dB high-noise conditions are summarized in Table 1. A horizontal comparison of the evaluation parameters reveals that although the model performance experienced a predictable slight decline under 20 dB strong background noise interference, the overall accuracy remained stable at or above 95.71%, reflecting the model’s strong robustness to noise interference. More importantly, the model performed excellently in both scenarios for normal operation, directly demonstrating the algorithm’s robustness in complex environments. This effectively avoids unnecessary actions caused by misjudgments in industrial settings, providing a reliable decision-making basis for the intelligent operation and maintenance of H-SSTS in real power distribution scenarios.

## 4. Experimental Verifications

### 4.1. Experimental Platform

**Figure 8:**
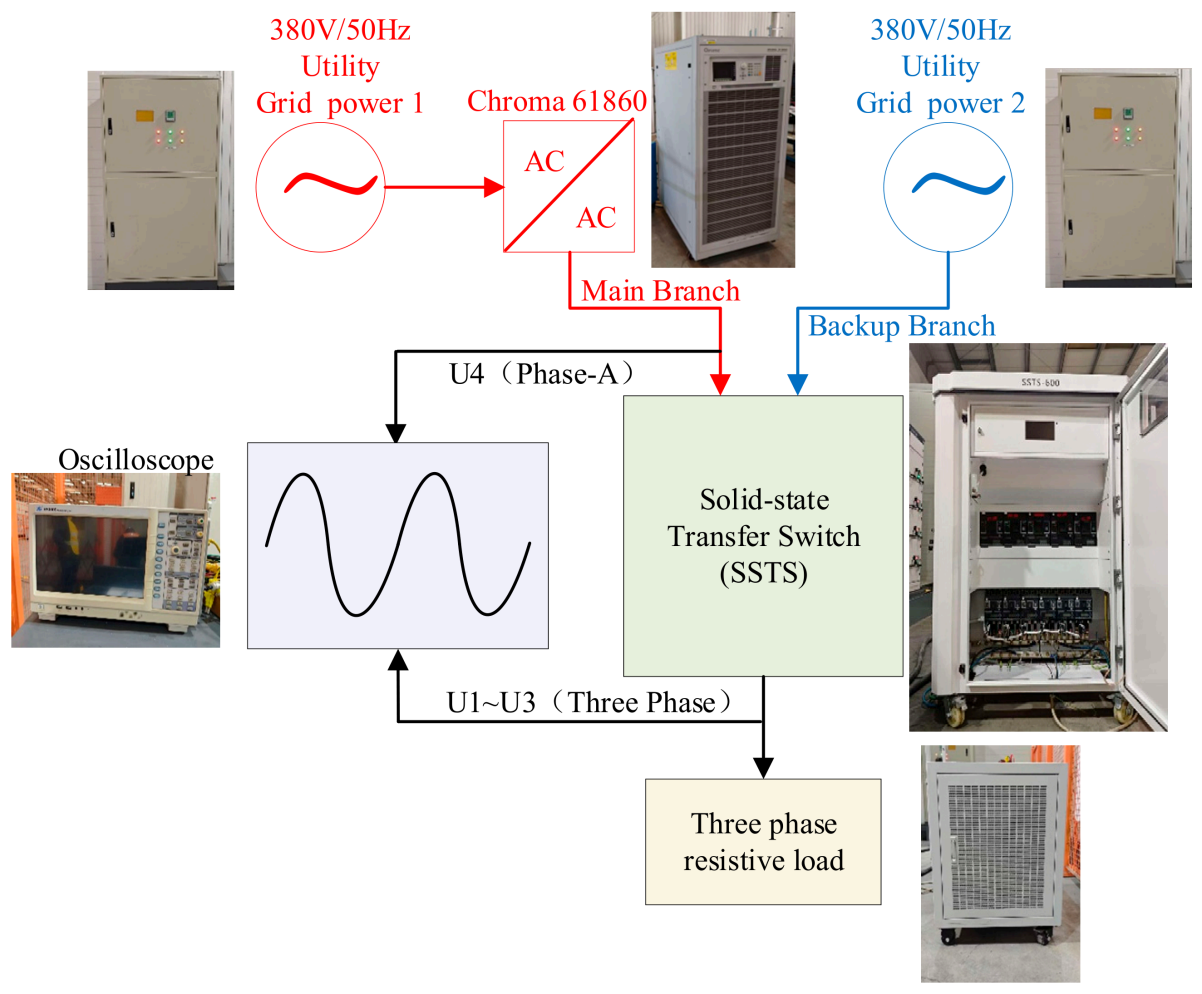
Experimental test platform topology.

To evaluate the switching performance of the proposed hybrid SSTS under representative distribution-grid operating conditions, a physical prototype test platform was established, as shown in Figure 8. The platform emulates a dual-source distribution system and consists of a programmable main source, a backup source, the proposed SSTS prototype, a waveform recorder, and a controllable load. On the main-source side, a Chroma 61860 regenerative grid simulator (Chroma ATE Inc., Taoyuan, Taiwan) is used to reproduce a three-phase 50 Hz grid and generate representative voltage disturbances, including undervoltage, overvoltage, and complete voltage interruption. The backup-source side is supplied by a three-phase 50 Hz utility source. A balanced three-phase resistive load bank is connected at the load side to provide controllable and repeatable load conditions. During each test, the A-phase voltage of the main source (U4) and the three-phase load-side voltages (U1–U3) are recorded synchronously. The transfer time is defined as the interval from the onset of a voltage disturbance in the main source to the restoration of load supply by the backup source. This interval includes disturbance detection, main-source disconnection, and backup-source connection. The subsequent closing of CK2 and the associated 30 ms delay occur after the backup source has taken over the load and are therefore excluded from the transfer time. Further source-transfer tests at higher load currents are required to evaluate current commutation, interruption capability, transient stresses, and thermal performance.

### 4.2. Experimental Verification of the Commutation Process and Switching Timing

**Figure 9:**
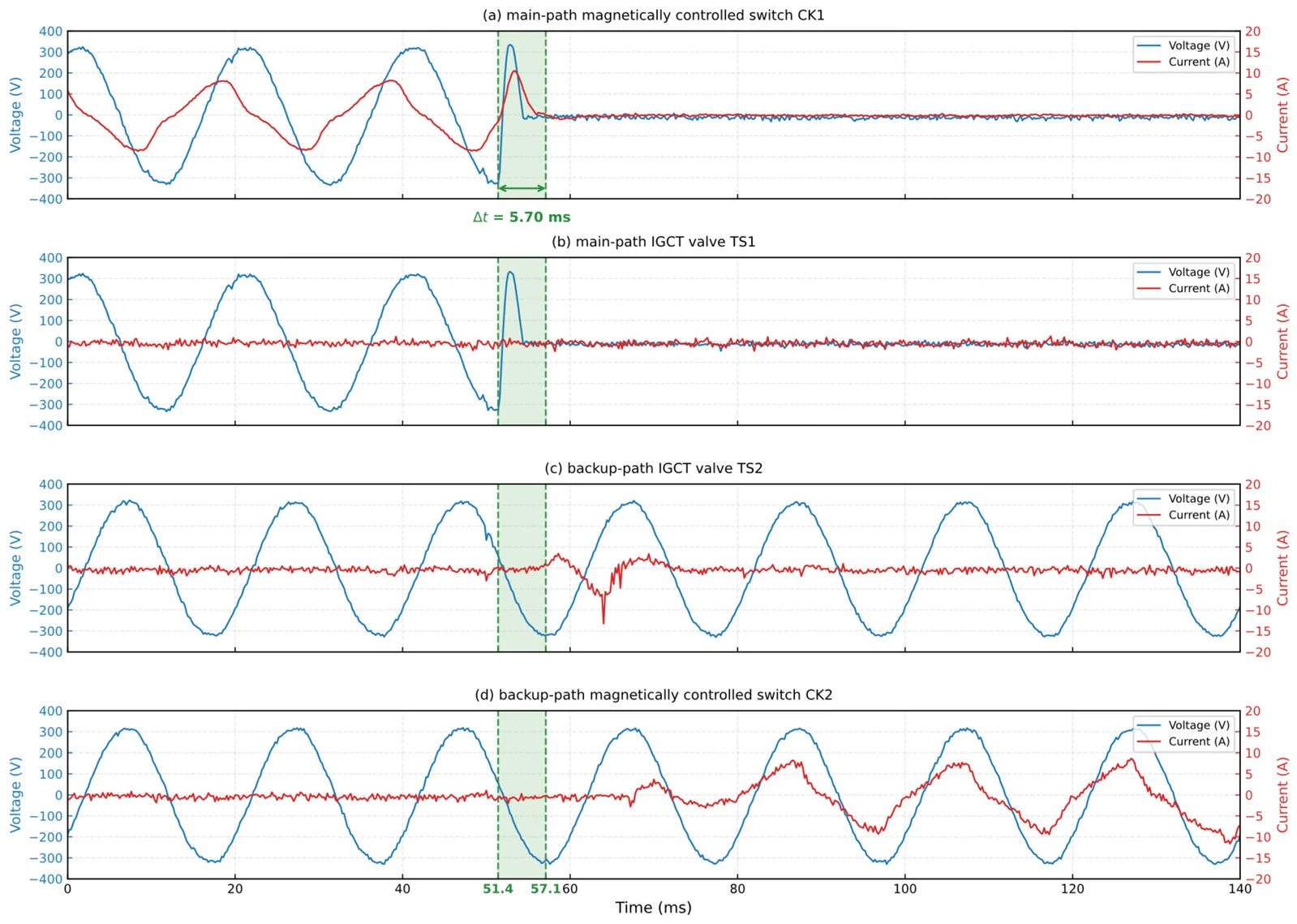
Voltage and current waveforms of the single-phase transfer test.

To verify the commutation mechanism described in Section 2.2 and the switching control logic described in Section 2.3, this paper conducts a single-phase power supply switching experiment. The voltages across the main circuit magnetic switch CK1, the main circuit IGCT valve body TS1, the backup circuit IGCT valve body TS2, and the backup circuit magnetic switch CK2, as well as the current flowing through each device, were measured. The experimental results are shown in Figure 9.

Under normal operating conditions, the load current is primarily carried by the main circuit magnetic switch CK1. When a transfer request is generated, the controller first enables TS1 to prepare the parallel commutation path and then issues an opening command to CK1. As the CK1 contacts separate under nonzero current, a brief vacuum arc develops and its voltage drives current commutation into TS1. The current through the mechanical contacts consequently decreases, limiting their arcing burden. After CK1 opens, the controller issues a turn-off command to TS1; as a fully controllable device, TS1 is capable of interrupting the current within a hundred microseconds. Subsequently, the system determines that the main circuit has been fully isolated and permits the activation of the backup power supply.

After confirming complete isolation of the main circuit, the controller triggers the backup circuit IGCT valve body TS2, establishing a temporary power supply channel for the backup power supply. After TS2 establishes a stable current flow, the controller sends a closing command to the standby magnetic switch CK2, while simultaneously keeping TS2 in the conducting state. Considering the physical bounce of the mechanical contacts at the moment of closing, the system turns off TS2 after a 30 ms delay. The load current then transfers from the TS2 branch to the CK2 branch, and ultimately CK2 bears the steady-state load current. Therefore, the complete switching control process sequentially includes TS1 triggering, CK1 opening, TS1 turning off, main circuit current threshold judgment, TS2 triggering, CK2 closing, a 30 ms delay, TS2 turning off, and the transfer of load current to CK2. This process is consistent with the switching control logic shown in Figure 2.

It should be noted that the current measurements shown in Figure 9c,d exhibit apparent amplitude deviations during the switching process, which may be associated with the dynamic response and settling time of the measurement equipment. However, the precise causes and magnitudes of these deviations have not been independently verified. The synchronous voltage waveforms provide supporting evidence for the switching sequence, but they do not allow the actual transient current amplitudes to be determined reliably. Therefore, the current waveforms are used only to provide qualitative information on the switching sequence and are not used to quantify transient current peaks or commutation dynamics.

### 4.3. Typical Disturbance Condition Switching Waveform Analysis

**Figure 10:**
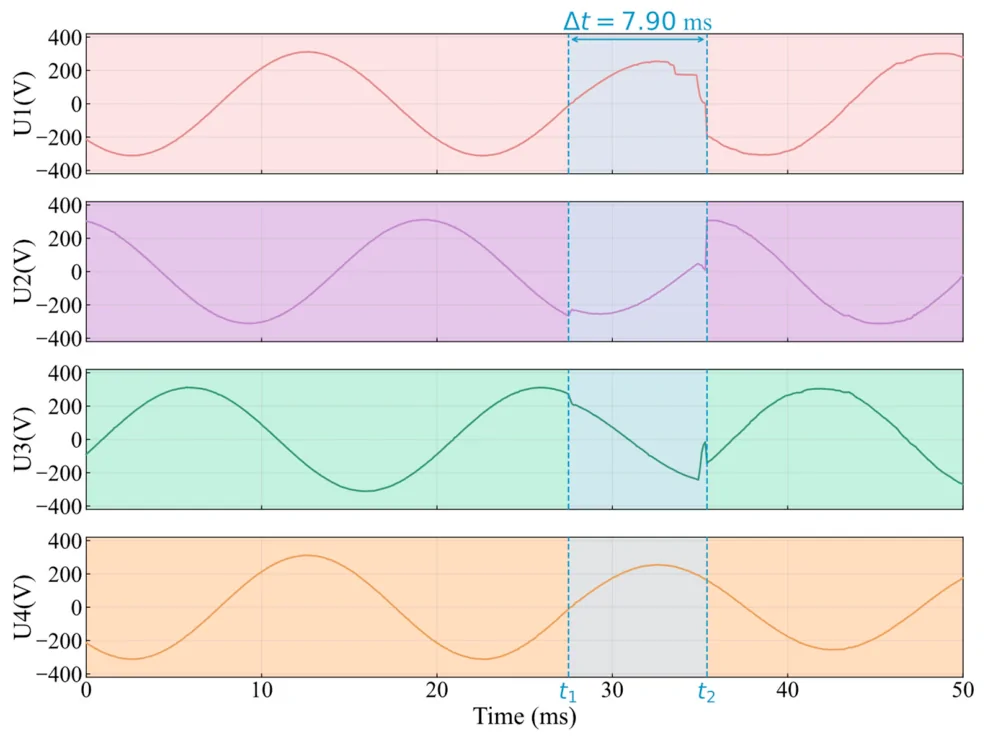
Experimental waveforms of a 0.8 p.u. undervoltage.

Amplitude-type faults are the most common fault type in distribution networks, placing the highest demands on hardware disconnection capabilities. Therefore, this paper selects two amplitude-type faults—interruption and undervoltage—for equipment performance evaluation. Figure 10 shows the experimental voltage waveform change when the main line voltage (U4) drops to 0.8 per unit with a total switching time of 7.90 milliseconds. As shown in the figure, when the main line voltage drops, the dq transform detection algorithm can immediately detect subtle deviations in the voltage vector and drive subsequent hardware coordination actions, indicating that the detection algorithm has extremely high sensitivity.

**Figure 11:**
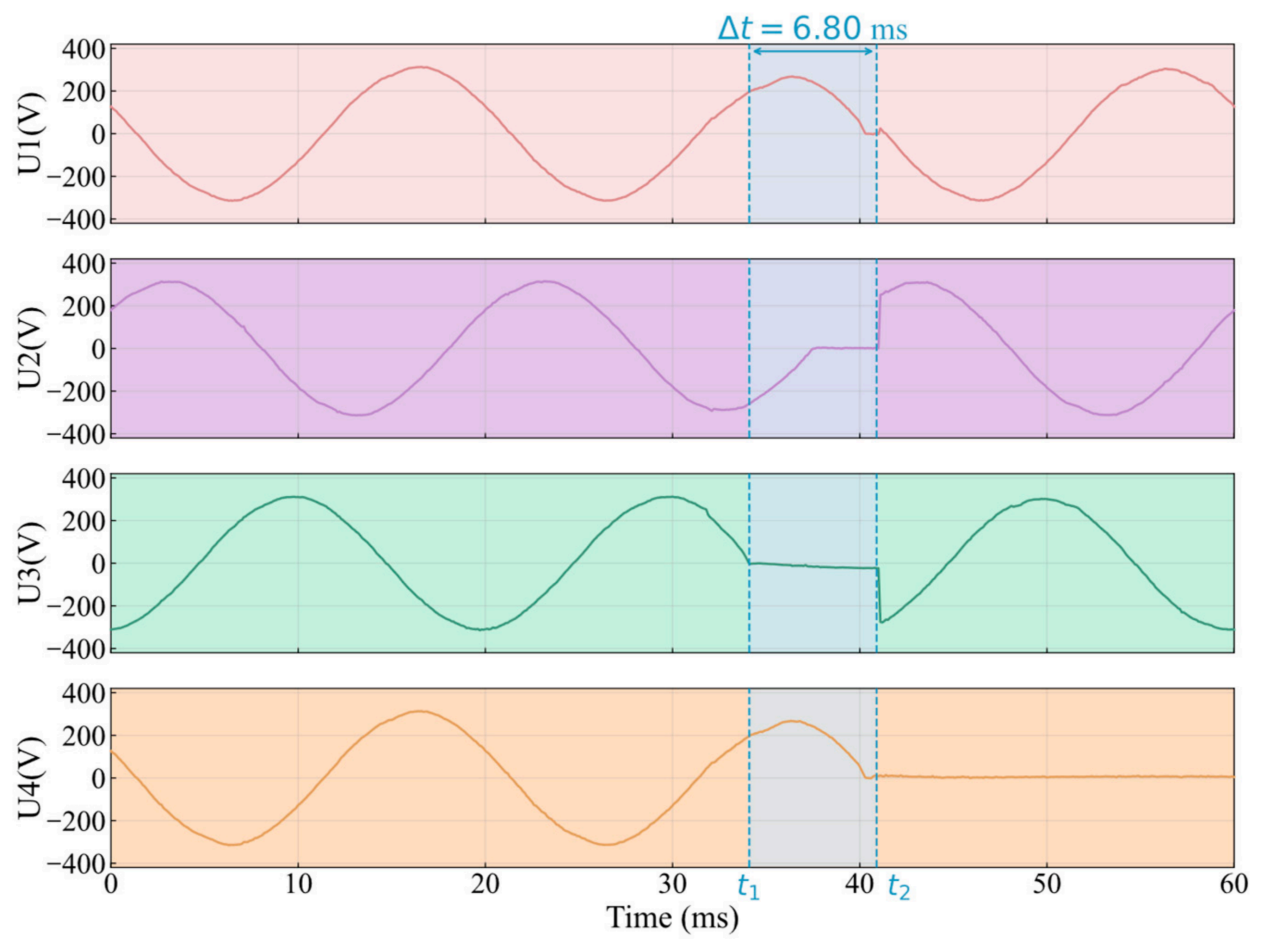
Experimental waveforms of full-voltage interruption.

Figure 11 shows the experimental voltage waveform when the main circuit voltage drops to 0 p.u. Under this condition, the voltage vector offset is more significant. The detection algorithm can more quickly and accurately capture the fault characteristics, and the device switching time is further shortened to 6.80 ms.

### 4.4. Performance Under Representative Voltage-Disturbance Conditions

More than 1200 transfer trials were conducted under the tested operating conditions. All recorded trials completed the transfer to the backup source and restored the load supply. Here, a successful transfer refers to completion of this operation and does not imply that every transfer was completed within 9 ms.

Using the transfer-time definition given in Section 4.1, the mean transfer time across all recorded trials was approximately 9 ms. With the IGCT valve branches functioning normally, the maximum observed transfer time was approximately 12 ms. A separate test condition simulated valve-branch unavailability, under which transfer was performed using only the permanent-magnet-actuated mechanical switches. The longest observed transfer time occurred under this condition and was approximately 20 ms.

The transfer times presented in Section 4.3 characterize the illustrated disturbance cases. The approximately 20 ms result characterizes mechanical-only transfer under the simulated valve-branch failure condition and should be distinguished from the normal hybrid transfer performance. The reported statistics apply to the tested load levels and operating conditions.

The experimental validation was limited to relatively low-current laboratory conditions. The reported transfer times should therefore be interpreted within this tested operating range. Further experiments at higher load currents are required to establish the current commutation, interruption, and thermal performance of the proposed system for distribution-network applications.

### 4.5. Performance Comparison Experiment of Various Changeover Switches

**Figure 12:**
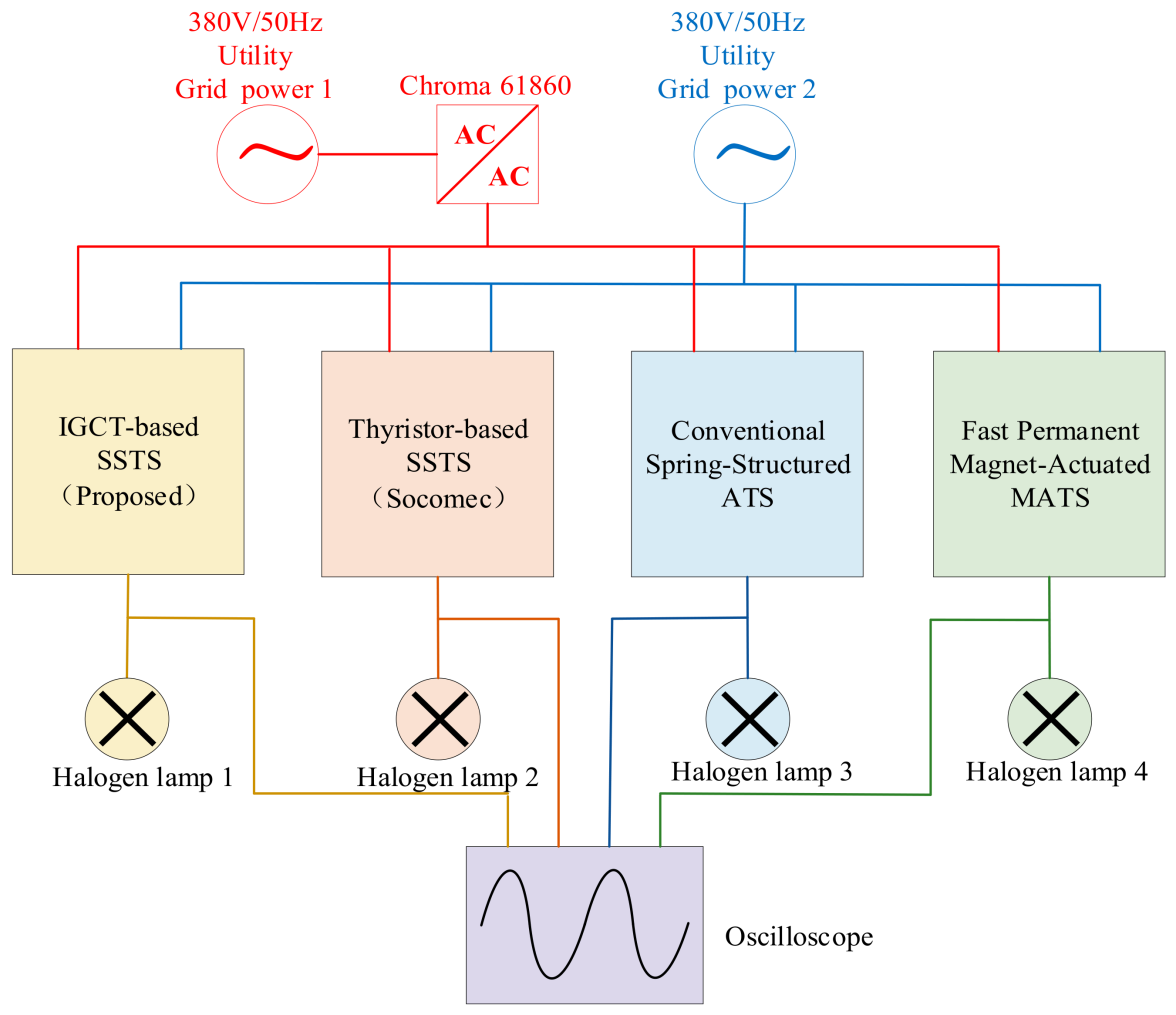
Transfer switch performance comparison experiment topology.

To visually highlight and compare the switching performance of this device with mainstream switching devices currently on the market, a comparative experiment as shown in Figure 12 was designed. The main source was provided by a Chroma 61860 programmable grid simulator, which generated a controlled voltage interruption, while the backup source was supplied from the utility grid. Four transfer devices were evaluated: a conventional mechanical automatic transfer switch (ATS), a magnetic automatic transfer switch (MATS), a commercial thyristor-based solid-state transfer switch (Socomec SSTS; Socomec SAS, Benfeld, France), and the proposed H-SSTS. Each device supplied an identical lamp load connected to its phase-A output. Under normal operating conditions, halogen lamps will extinguish directly when a power outage exceeds their threshold time. Even if power is immediately restored after extinguishing, it takes 5–6 min to regain normal brightness, which effectively reflects the differences in switching time between the devices. The same source, load, and interruption settings were applied to all four devices. Because only one phase was used, this experiment evaluates single-phase transfer performance.

**Figure 13:**
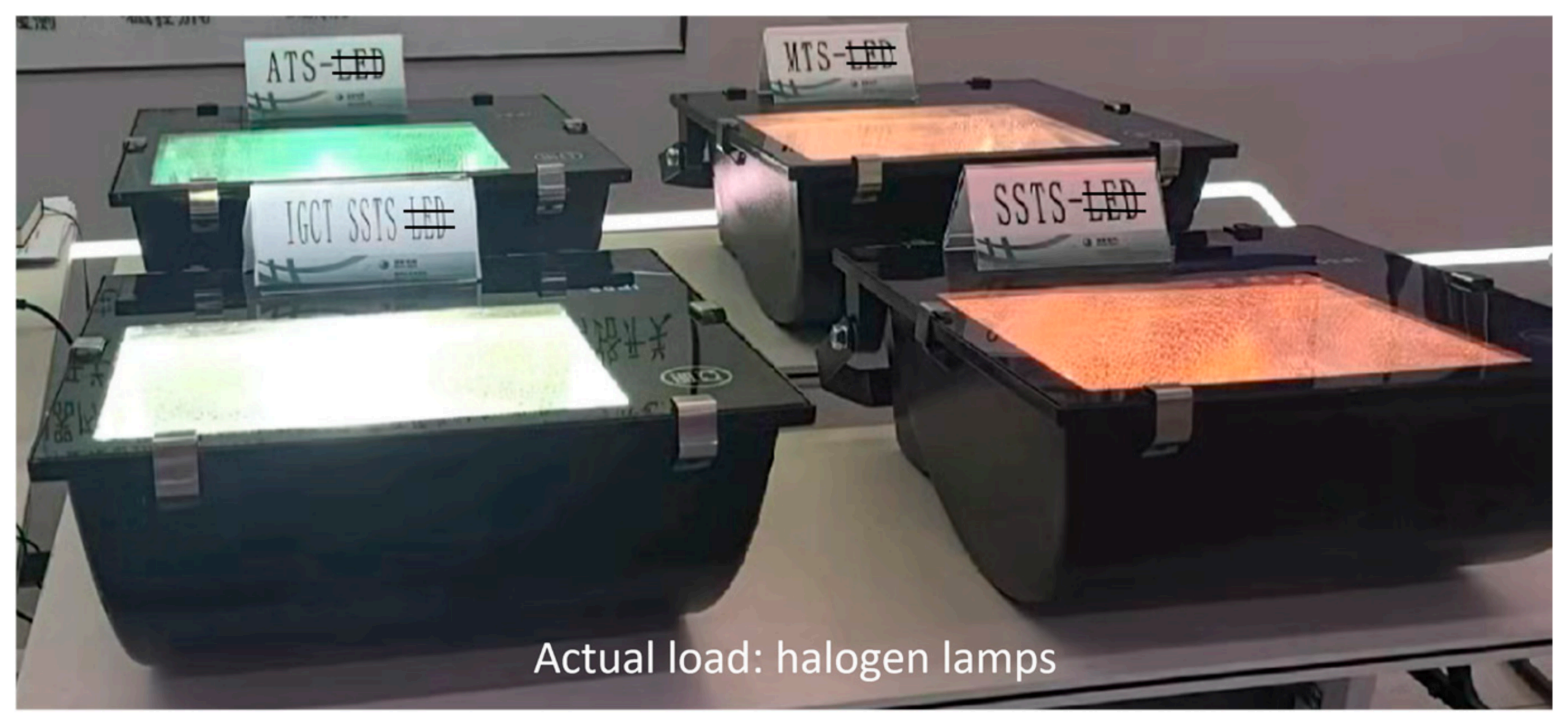
Comparison of lamp brightness during power transfer using different switching devices. The incorrect “LED” designations on the placards have been crossed out, and an annotation identifying the actual loads as halogen lamps has been added during revision.

An interrupt signal was uniformly sent to the main circuit of each device using a Chroma 61860, and the response of the halogen lamps connected to each device was observed. The experiment showed that the halogen lamps connected to the mechanical automatic transfer switch (ATS) and the magnetic automatic transfer switch (MATS) went out directly, indicating that their switching time exceeded the halogen lamp’s power-off tolerance threshold. The lamps connected to the Socomec SSTS and the device studied in this paper did not go out. Slow-motion playback of the experiment using a camera, as shown in Figure 13, revealed that the halogen lamp connected to the Socomec SSTS dimmed during switching, while the halogen lamp connected to the device studied in this paper remained bright throughout. This indicates that the switching time of the device studied in this paper is shorter than that of the Socomec SSTS.

**Figure 14:**
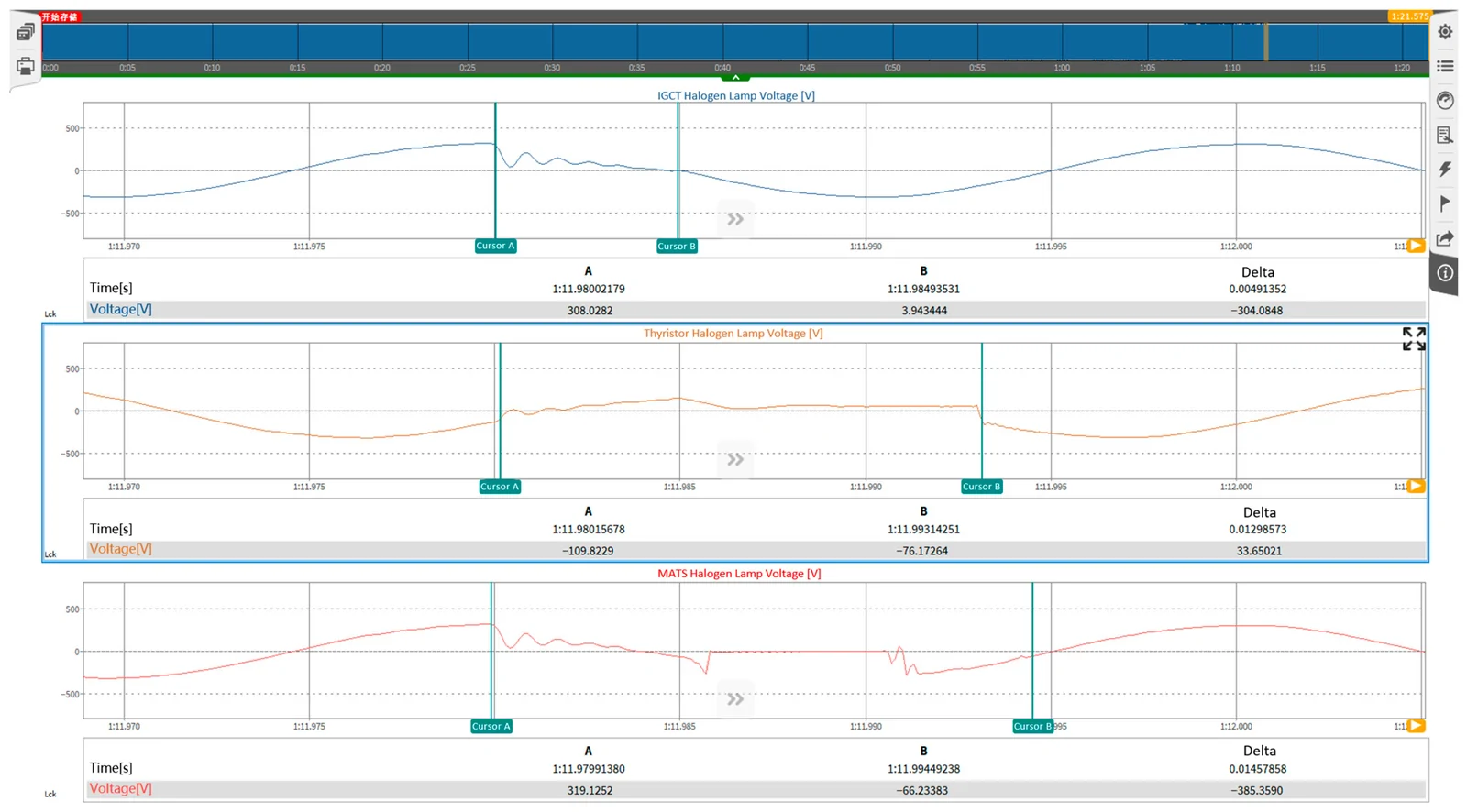
Comparison of switching times of three typical changeover switches.

Figure 14 shows the waveform changes before and after switching for the three devices in the experiment. The Automatic Transfer Switch (ATS) has the longest switching time and is therefore not shown in the figure. As can be seen from the figure, although the MATS uses a magnetically controlled circuit breaker, it requires a significant amount of time to process the arc voltage generated during switch separation due to the lack of commutation coordination with power electronic devices, resulting in an overall switching time of 14.58 ms. The Socomec SSTS, using a thyristor-based SSTS, requires time to wait for the current to naturally cross zero, resulting in an overall switching time of 12.99 ms. In contrast, the device developed in this paper requires a switching time of only 4.91 ms, demonstrating significantly superior switching performance compared to the other three mainstream switching devices on the market. Under the specified single-phase test conditions, the proposed H-SSTS achieved the shortest measured transfer time, which was approximately 62.2% shorter than that of the Socomec SSTS.

### 4.6. Typical Load Compatibility and Real-World Testing

To verify the reliability of the prototype under real power grid operation conditions, this paper selected a power-type inductive device and an information-sensitive load for on-site grid connection testing.

The power-type inductive device was a 3 kW single-phase asynchronous motor. The motor was connected to the load side of the device, and a transfer test was performed. Its operation was observed before and after the transfer. During the experiment, the machine’s operating sound remained stable throughout, without significant speed fluctuations or triggering of the trip protection. This indicates that the orderly integration between the IGCT’s turn-off sequence and the absorption circuit effectively mitigated the back EMF impact during inductive current interruption.

Subsequently, an information-sensitive load connection test was conducted. Two personal computers were connected to the outgoing line. When the programmable power supply repeatedly generated disturbances such as power outages and temporary voltage sags, the tested computers did not restart or display a black screen, and no operating data was lost.

## 5. Conclusions

This study presented an IGCT-based hybrid solid-state transfer switch (H-SSTS) incorporating magnetically controlled switches for voltage-disturbance identification and fast transfer control in distribution systems supplying sensitive loads. The proposed system combines a hybrid switching topology with a hierarchical control architecture. A dq-transform-based triggering strategy is used to rapidly detect abnormal voltage conditions and initiate the transfer process, while a deep neural-network model with multiscale feature fusion identifies disturbance conditions from transient voltage-waveform features.

The proposed method was evaluated through simulation and physical prototype tests. At a signal-to-noise ratio of 20 dB, the diagnostic model achieved fault-identification accuracies of 99.86% on the source dataset and 95.71% on the generalization dataset. In single-phase full-voltage-interruption tests, the proposed H-SSTS achieved a transfer time of 4.91 ms, which was approximately 62.2% lower than that of the tested commercially available Socomec static transfer switch under the same test conditions. In three-phase full-voltage-interruption tests, the proposed prototype achieved a transfer time of 6.80 ms. Additional tests with personal computers and asynchronous motors verified stable load transfer during the evaluated voltage disturbances.

These results demonstrate that the proposed H-SSTS integrates voltage-disturbance identification with rapid transfer control under the tested conditions. The experimental validation of source-transfer performance is limited to the relatively low-current conditions examined in this study. Further tests at higher load currents are required to evaluate current commutation, interruption capability, transient voltage and current stresses, and thermal performance. Future work will also investigate a broader range of grid disturbances and load types.

## Figures and Tables

**Table 1:** Classification Performance Evaluation of the Intelligent Diagnostic Algorithm.

Dataset	Overall Accuracy (%)	Macro Recall (%)	Macro F1-Score (%)	Normal-Class Precision (%)
Source dataset (overall)	99.96	99.96	99.96	99.75
Source dataset (20 dB)	99.86	99.86	99.86	99.01
Generalization dataset (overall)	97.91	97.91	97.91	99.80
Generalization dataset (20 dB)	95.71	95.71	95.72	99.01

## Data Availability

The data presented in this study are available from the corresponding author upon reasonable request.

## Abbreviations

The following abbreviations are used in this manuscript:

AC	Alternating Current
ATS	Automatic Transfer Switch
DNN	Deep Neural Network
EMD	Empirical Mode Decomposition
FFT	Fast Fourier Transform
HHT	Hilbert–Huang Transform
H-SSTS	Hybrid Solid-State Transfer Switch
IGBT	Insulated-Gate Bipolar Transistor
IGCT	Integrated Gate-Commutated Thyristor
IMF	Intrinsic Mode Function
ITIC	Information Technology Industry Council
MATS	Magnetically Controlled Automatic Transfer Switch
PCA	Principal Component Analysis
SNR	Signal-to-Noise Ratio
SCR	Silicon-Controlled Rectifier
THD	Total Harmonic Distortion
WPT	Wavelet Packet Transform
